# Management of Warfarin-Induced Adrenal Adenoma Hemorrhage in the Setting of a History of Pulmonary Embolism

**DOI:** 10.7759/cureus.32556

**Published:** 2022-12-15

**Authors:** Masha Osman, Maria J Kowzun, James E Gervasoni

**Affiliations:** 1 Department of General Surgery, School of Medicine, St. George's University, New York, USA; 2 Department of Surgery, Robert Wood Johnson University Hospital, New Brunswick, USA; 3 Department of Surgery, Saint Peter’s University Hospital, New Brunswick, USA

**Keywords:** adrenalectomy, anticoagulation, warfarin, adrenal adenoma, adrenal hemorrhage

## Abstract

Adrenal hemorrhage (AH) is associated with trauma, acute stress, sepsis, coagulopathy, pregnancy, neonatal stress, and underlying adrenal masses, which can include metastases, pheochromocytomas, adrenocortical cancers, or hematomas. However, the literature on nontraumatic AH secondary to an adenoma in the setting of chronic anticoagulation is limited. We present a case report of a patient found to have AH from an incidental adrenal adenoma associated with the use of warfarin in the setting of a recent history of pulmonary embolism requiring anticoagulation. In a patient who presents with AH while on anticoagulation, initial management should include reversal of coagulopathy, supportive care with serial hematocrits and blood transfusions as necessary, and biochemical workup to assess for functional tumors. However, aggressive surgical management with adrenalectomy should ideally follow for those patients who will require long-term anticoagulation to minimize future risk for rebleeding.

## Introduction

Adrenal hemorrhage (AH) is associated with trauma, acute stress, sepsis, coagulopathy, pregnancy, neonatal stress, and underlying adrenal masses, which can include metastases, pheochromocytomas, adrenocortical cancers, or hematomas [1,2]. In cases of trauma and acute illness, the incidence of AH in autopsy studies ranges from 0.14% to 25% [1]. With the improvement of imaging technology in past years, AH has been diagnosed earlier in 1.5% to 5% of hospitalized patients [1]. However, the literature on nontraumatic AH secondary to an adenoma in the setting of chronic anticoagulation is limited. This is the first report describing the successful management of warfarin-induced hemorrhage of an incidental adrenal adenoma in a patient with a history of pulmonary embolism requiring chronic anticoagulation.

## Case presentation

**Figure 1:**
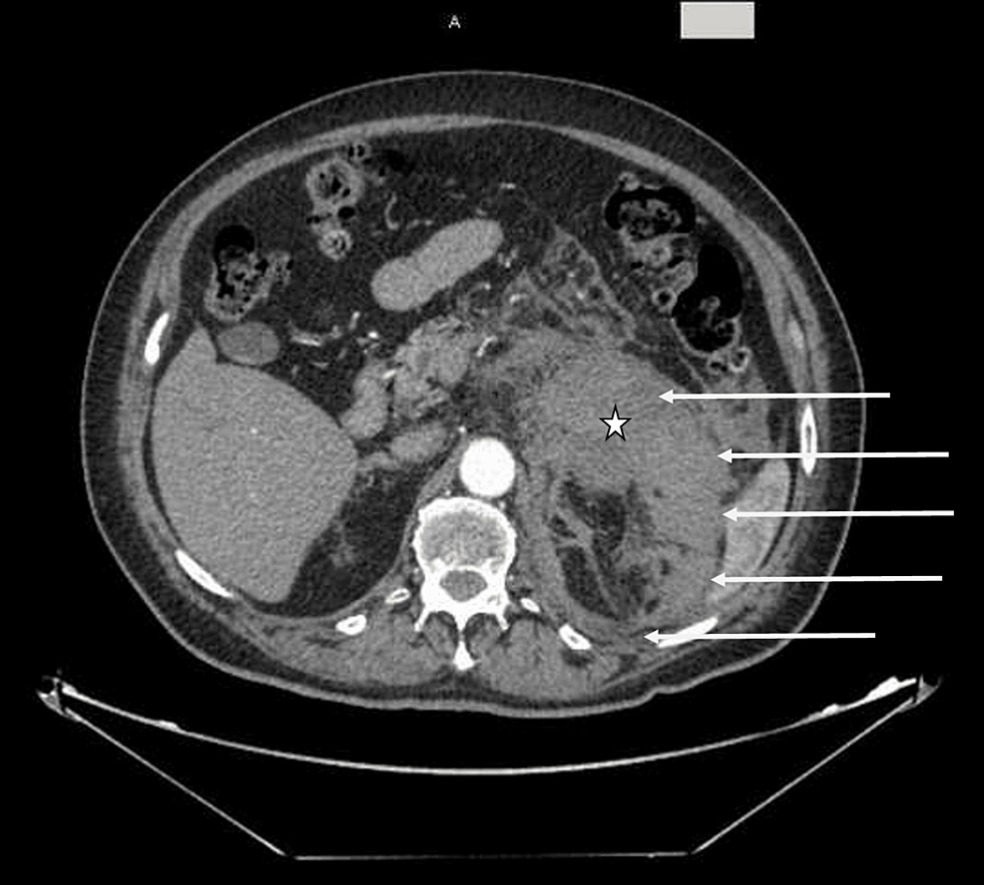
CT abdomen/pelvis showing an acute, moderate-sized left retroperitoneal hemorrhage (12 cm × 11.2 cm × 6.1 cm) centered around the left adrenal gland. The star and arrows delineate the bulk and outer borders of the adrenal hemorrhage, respectively. CT, computed tomography

A 74-year-old man with a history of diabetes, hypertension, hypothyroidism, benign prostatic hyperplasia, and a recent pulmonary embolism with warfarin treatment ongoing presented with complaints of left abdominal and flank pain for one day. On initial evaluation, he was found to have orthostatic hypotension, mild abdominal distension, and left-sided abdominal tenderness. He otherwise denied any fever, vomiting, chills, chest pain, or shortness of breath. Laboratory blood tests showed a hemoglobin level of 11.9 g/dL and an international normalized ratio (INR) of 2.7. Computed tomography (CT) scan of the abdomen and pelvis revealed an acute, moderate-sized left retroperitoneal hemorrhage (12 cm × 11.2 cm × 6.1 cm) centered around the left adrenal gland, most likely secondary to a hemorrhagic left adrenal mass (Figure 1). His warfarin was subsequently held, and he was treated with vitamin K and fresh frozen plasma. By the next morning, his hemoglobin level had dropped from 10.9 to 7.7 g/dL; he was transfused one unit of packed red blood cells and closely monitored. Over the next several days, serial hemoglobin levels remained stable between 7 and 8 g/dL.

**Figure 2:**
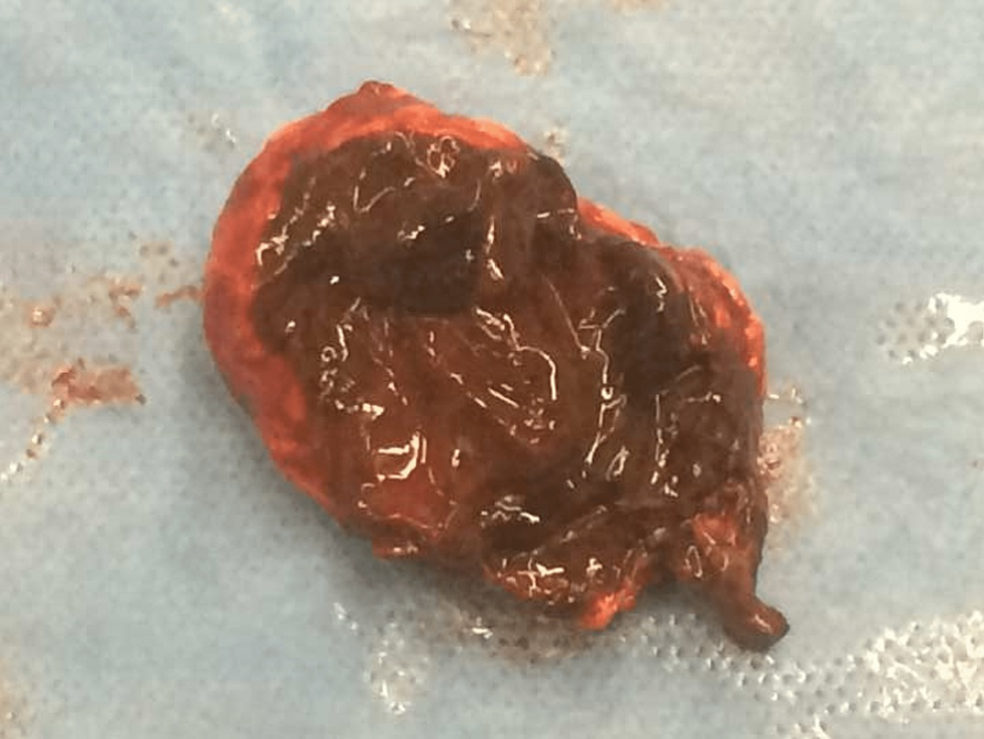
Pathology specimen of the adrenal mass.

On hospital day 6, he was taken to the operating room for elective removal of his left adrenal gland. After creating a left subcostal incision, a large amount of adhesions and clotted blood was immediately noted, and approximately 300 cc of hematoma was evacuated. Further dissection revealed a fractured adrenal gland with hemorrhage within the gland spilling into the retroperitoneum. The adrenal gland was removed and sent to pathology (Figure 2), with a frozen specimen positive for adrenal adenoma. The wound was closed in layers, and the patient tolerated the procedure well. He received two units of blood intraoperatively. On postoperative day 1, an inferior vena cava (IVC) filter was placed. His hemoglobin remained stable between 9 and 10 g/dL, and he was eventually discharged home three days after his surgery. Final specimen pathology eventually came back as an adrenal gland consistent with adrenal cortical adenoma and extensive hemorrhage with tumor infarction, as well as an adrenal capsule composed of fibroadipose tissue with hemorrhage.

## Discussion

**Figure 3:**
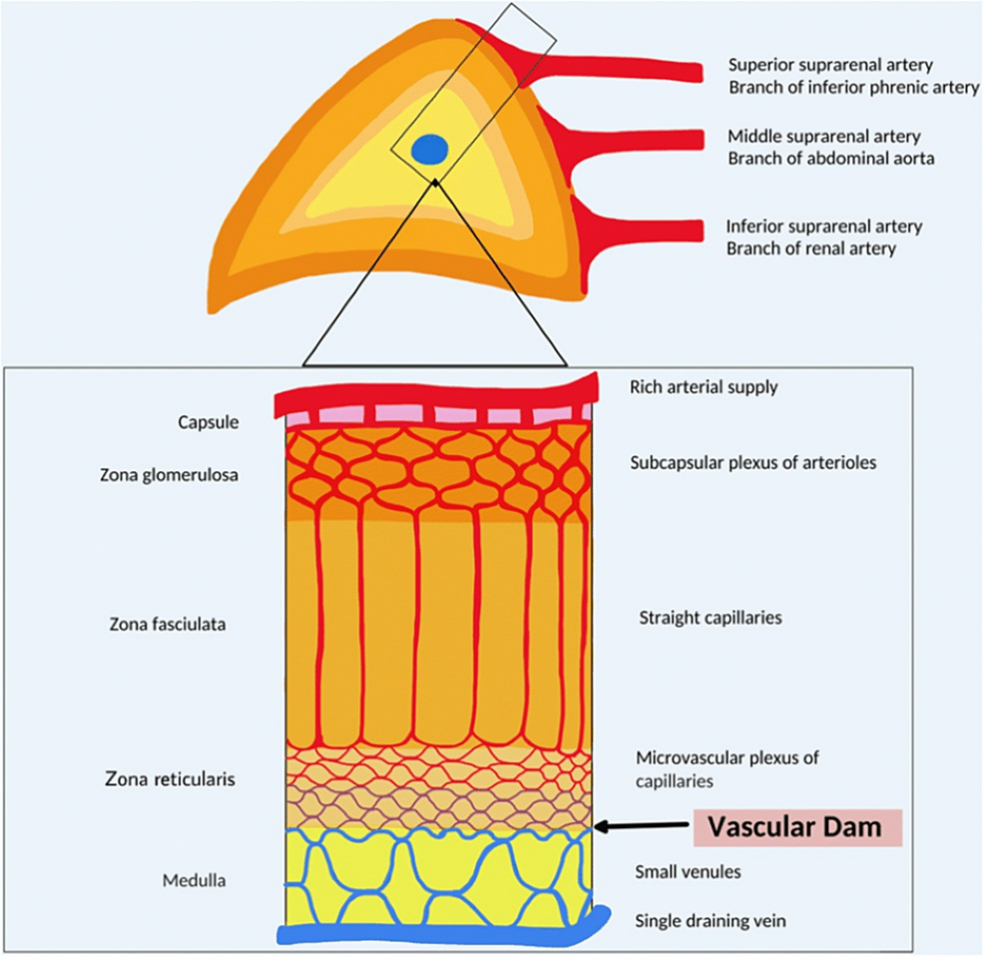
Vascular anatomy of the adrenal gland. Source: [4]

The highly vascular anatomy of the adrenal gland supplied by 50 to 60 small arterial branches from three suprarenal arteries makes it particularly susceptible to hemorrhage (Figure 3) [3,4]. In a literature review of 133 reported cases of spontaneous hemorrhage from adrenal masses, 80% of these cases were benign while 20% were malignant lesions [2]. Pheochromocytoma was the most common cause of bleeding from a primary adrenal tumor (48%), followed by metastatic lesions to the adrenal gland (14%), hematoma (13%), myelolipoma (10%), adrenocortical carcinomas (7%), adenoma (4%), pseudocyst/hematoma in pregnancy (4%), and lipoma (1%). Adrenal adenomas are common but rarely hemorrhage. However, lesions larger than 10 cm are at risk for spontaneous bleeding into the adrenal gland or retroperitoneum [1]. In other studies of patients requiring anticoagulation after bilateral knee arthroplasty and without adrenal masses, the use of anticoagulants such as heparin and warfarin increased the risk of adrenal hemorrhage by 5- to 10-fold [5]. Several cases of bilateral massive adrenal hemorrhage have been reported in the setting of heparin-induced thrombocytopenia (HIT) and subsequent paradoxical high risk of thromboembolism, leading to thrombosis of the central adrenal vein with ensuing adrenal hemorrhage [1].

Depending on the severity of the adrenal hemorrhage, the clinical presentation may include acute onset of abdominal or flank pain, nausea, vomiting, changes in blood pressure, palpable flank mass, agitation, mental status changes, and low-grade fevers [3]. Laboratory evaluation may reflect a drop in hemoglobin and occasional electrolyte imbalances secondary to adrenal insufficiency. Though various imaging modalities can be used, CT scans are the most common tests for diagnosing adrenal hemorrhage, with findings of periadrenal stranding or retroperitoneal and crural thickening. Other imaging modalities include ultrasound (preferred in neonates) and magnetic resonance imaging studies.

Based on the literature review, Marti et al. [2] suggested recommendations for the management of adrenal hemorrhage. If there is no other indication for abdominal exploration, nonoperative management consisting of supportive care, measurement of serial hematocrits, and administration of blood transfusions as needed is recommended. In addition, a biochemical workup should be performed to rule out a hormonally active tumor, with an evaluation of both glucocorticoid and catecholamine excess. In the setting of ongoing adrenal hemorrhage refractory to blood transfusions, endovascular embolization of the bleeding adrenal artery is preferred if interventional capabilities are available, as emergency surgery carries a mortality rate of 45% [1]. If the patient is hemodynamically stable and there is evidence of a functional tumor, then interval adrenalectomy can be performed in a delayed fashion to allow adequate time for the resolution of the hematoma and inflammatory response, thus minimizing morbidity. If the tumor is nonfunctional, interval imaging may be an option to monitor for the resolution of the hematoma, thus obviating the need for surgery.

## Conclusions

In this case, the patient was found to have adrenal hemorrhage from an incidental adrenal adenoma associated with the use of warfarin in the setting of a recent history of pulmonary embolism requiring anticoagulation. Initial management of the patient involved the reversal of his warfarin-induced coagulopathy and blood transfusion as needed. Although he remained hemodynamically stable with supportive care, his requirement for chronic anticoagulation was a known risk factor for future adrenal hemorrhage. Thus, he underwent surgical intervention for the removal of the large hematoma and the adrenal mass to minimize his likelihood of rebleeding. This case highlights the need to consider aggressive surgical management in patients requiring chronic anticoagulation who present with adrenal mass hemorrhage. Surgical intervention with the evacuation of the hematoma and adrenalectomy should be performed in these patients because of the increased risk for subsequent bleeding while on long-term anticoagulation. Appropriate follow-up regarding adrenal function and anticoagulation status should also be carried out once the surgery has been performed.
